# Comparison of adenoma detection rate between three-dimensional and standard colonoscopy: a multicenter randomized controlled trial

**DOI:** 10.1055/a-2510-8759

**Published:** 2025-02-28

**Authors:** Wei-Yuan Chang, Wei-Chih Liao, Li-Chun Chang, Hsuan-Ho Lin, Pin-Ya Wei, Hsing-Chien Wu, Han-Mo Chiu, Ming-Shiang Wu

**Affiliations:** 1 Department of Internal Medicine, National Taiwan University Hospital, Taipei, Taiwan; 2 Health Management Center, National Taiwan University Hospital, Taipei, Taiwan; 3 Internal Medicine, National Taiwan University College of Medicine, Taipei, Taiwan; 4 Department of Internal Medicine, National Taiwan University Hsinchu Branch, Hsinchu, Taiwan; 5 Department of Internal Medicine, National Taiwan University Cancer Center, Taipei, Taiwan

## Abstract

**Background:**

Improvement in adenoma detection rates (ADRs) effectively reduces the incidence of colorectal cancer. In a simulation study, three-dimensional (3D) colonoscopy provided more anatomical details than standard two-dimensional (2D) colonoscopy and improved ADR. We compared ADRs between 2D and 3D colonoscopy.

**Methods:**

In this multicenter randomized controlled trial, participants aged ≥40 years undergoing colonoscopy for screening, surveillance, or symptoms between February 2022 and June 2023 were randomized 1:1 into 2D or 3D groups. The primary outcome was ADR. Secondary outcomes included detection rates for flat adenomas, right-sided adenomas, proximal adenomas, advanced adenomas, and sessile serrated lesions (SSLs).

**Results:**

Of 348 participants recruited, data from 158 (2D group) and 160 (3D group) were analyzed. Mucosa inspection time was comparable between the 3D (9.8 [SD 2.6] minutes) and 2D (9.4 [SD 3.1] minutes) groups ( *P* = 0.21). Compared with the 2D group, the 3D group had a significantly higher ADR (53.1% vs. 38.6%; difference 14.5% [95%CI 3.7 to 25.4]; *P* = 0.009), and higher detection rates for flat adenomas (35.0% vs. 21.5%; difference 13.5% [95%CI 3.7 to 23.3]; *P* = 0.008), right-sided adenomas (26.3% vs. 15.2%; difference 11.1% [95%CI 2.2 to 19.9]; *P* = 0.02), proximal adenomas (38.1% vs. 23.4%; difference 14.7% [95%CI 4.7 to 24.7]; *P* = 0.005), and adenomas sized 5–9 mm (45.0% vs. 31.0%; difference 14.0% [95%CI 3.4 to 24.5]; *P* = 0.01). There was no difference in detection rates for SSLs or advanced adenomas.

**Conclusion:**

3D colonoscopy improved adenoma detection without significantly increasing the mucosa inspection time.

## Graphical abstract

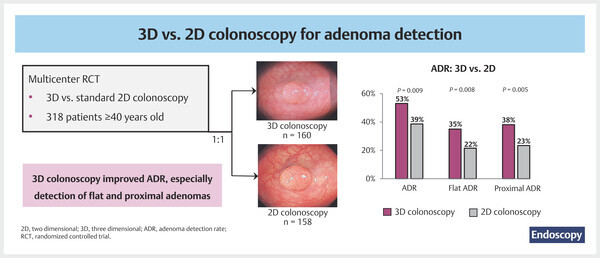

## Introduction

Colorectal cancer (CRC) is the third most common cancer and the second leading cause of cancer-related deaths worldwide 1 . Most sporadic CRCs arise from pre-existing adenomas 2 , and removal of these precancerous lesions has been shown to effectively reduce both the incidence and mortality of CRC 3 4 . Therefore, the effectiveness of colonoscopy in protecting against CRC hinges on the detection and removal of adenomas, and the adenoma detection rate (ADR) is the most important quality indicator of colonoscopy. Previous research showed that a 1% increase in ADR can reduce CRC incidence and mortality by 3% and 5%, respectively 5 . Therefore, various modalities have been developed to improve ADR, including image-enhancing technologies 6 , chromoendoscopy 7 , and devices enhancing exploration of the mucosa 8 9 .

Despite improvements in ADR conferred by those modalities, post-colonoscopy colorectal cancer (PCCRC) remains a concern 10 . The incidence of PCCRC has been reported at 8.6% within 3 years 11 , with more than 80% of PCCRCs being attributed to missed adenomas 12 13 . Notably, flat and proximal adenomas are independently associated with the development of PCCRC and are particularly difficult to detect, posing significant challenges to improving the ADR 12 14 15 .

Three-dimensional (3D) endoscopy provides 3D visualization with superior depth perception over conventional two-dimensional (2D) endoscopy and may thereby enhance detection of flat/superficial lesions and subtle mucosal changes. 3D endoscopy has shown promise in enhancing the detection of superficial gastric neoplasms and accuracy in assessing morphology 16 . It has also been proposed that 3D endoscopy enhances the detection of colonic adenomas, showing a 25% increase in adenoma detection in a study using simulated 3D colonoscopy in a synthetic colon model 17 18 . However, whether 3D colonoscopy could improve ADR and facilitate detection of flat polyps compared with standard 2D colonoscopy in clinical colonoscopy practice remains to be studied.

The MonoStereo 3D endoscopic visualization system (MedicalTek Co. Ltd, Taichung, Taiwan) is a novel 3D endoscopy system that performs real-time conversion of standard 2D images to realistic 3D visualization during endoscopy and has been approved for clinical use 19 20 . We hypothesized that the 3D endoscopic visualization system could enhance polyp detection during colonoscopy, especially for flat/superficial polyps. Therefore, we conducted a randomized controlled trial (RCT) to investigate whether 3D colonoscopy improved adenoma detection compared with standard 2D colonoscopy.

## Methods

### Study design

This was a prospective, multicenter, randomized, open-label, single-blind trial conducted in one referral center and two regional hospitals in Taiwan. Complying with the principles of the Declaration of Helsinki and Good Clinical Practice guidelines, this trial was approved by the institutional review board of National Taiwan University Hospital (No. 202109112DIPB). An independent data and safety monitoring committee monitored the progress of the trial, with regular assessment of safety outcomes, overall trial integrity, and trial performance.

### Participants

Individuals aged 40 years or older who were scheduled for colonoscopy for screening, surveillance, or symptoms at outpatient clinics in the participating institutions were consecutively assessed for eligibility. Individuals with a contraindication to colonoscopy or polypectomy, or with a history of inflammatory bowel disease or hereditary polyposis syndrome were excluded.

### Randomization and masking

In this study, randomization was conducted centrally at the coordinating hospital by a research assistant using a computer-generated randomization sequence with a block size of 20, assigning participants from all three hospitals in a 1:1 ratio without stratification. Allocation concealment was ensured by storing the group allocation in ordered, sealed, and opaque envelopes. The patients and research assistants who assessed the outcomes were blinded to the group allocation to avoid bias.

### Procedures

#### Three-dimensional colonoscopy

Two- and three-dimensional colonoscopy. 2D images are converted to left/right images, which yield 3D images using 3D monitors and polarized glasses. Deliberate endoscope movement demonstrates minimal time lag. The apparent difference in polyp shape on 3D images disappears with the use of 3D monitors and polarized glasses.Video 1

**Fig. 1:**
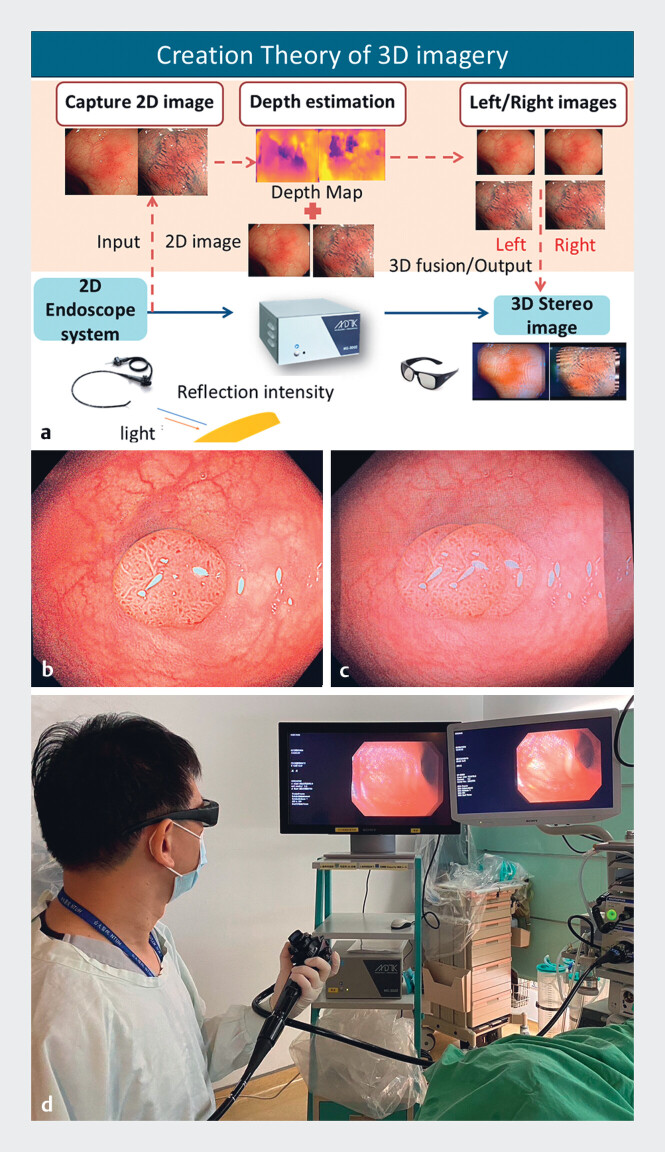
Use of the novel three-dimensional (3D) endoscopy system during colonoscopy. **a** Schematic representation of the MonoStereo 3D endoscopic visualization system (MedicalTek Co. Ltd, Taichung, Taiwan). **b,c** The endoscopic display can be switched from standard 2D images ( **b** ) to reconstructed images, which transform into real-time fully immersive 3D images when viewed with polarized 3D glasses ( **c** ). **d** Employing 3D colonoscopy during routine colonoscopic examinations. Source for Figure 1a: MedicalTek Co., Ltd.

Fig. 1**a** illustrates the MonoStereo 3D endoscopic visualization system. 2D images ( Fig. 1 **b** and right screen in Fig. 1 **d** ) are converted in 80 milliseconds to 3D images ( Fig. 1 **c** and left screen in Fig. 1 **d** ) visualized through polarized glasses worn by the endoscopist, providing real-time 3D imagery without perceptible time lag ( Fig. 1 **d** , Video 1 ). The system offers three pupillary distance selections to mitigate eye strain, and endoscopists are recommended to identify the optimal personal selection before first use by finding the selection yielding the most vivid 3D image. The system does not require calibration before examination; endoscopists are advised to place the 3D screen at eye level and stand in front of the screen at a distance tailored to individual preference (generally 100–150 cm for a 31”/32” screen). Instantaneous switch between 3D and standard 2D displays is achieved by pressing a button. As the polarized glasses do not change the visual perception of the surrounding environment or standard 2D endoscopic images, the endoscopist does not need to remove the glasses when not using the 3D display.

#### Intervention and colonoscopy

Study colonoscopies were performed by three junior colonoscopists (experience <5000 colonoscopies) and one senior colonoscopist (experience ≥5000 colonoscopies). Before the commencement of the study, the participating colonoscopists received an introduction on the 3D technology and equipment, and performed 3D colonoscopy using a colonoscopy simulator. Each colonoscopist was then requested to use 3D colonoscopy in conjunction with standard 2D colonoscopy for mucosa inspection during colonoscope withdrawal in at least 10 colonoscopic procedures Fig. 1 **d** ).

For the RCT, high-definition colonoscopes (290 series; Olympus, Tokyo, Japan) and video processors (EVIS Lucera Elite; Olympus) were used for colonoscopy. Bowel preparation and image-enhanced endoscopy were performed in the same way in both groups. Standard 2D colonoscopy was used for colonoscope insertion, as in routine clinical practice, in both groups. The use of distal attachment devices, such as cap or cuff, was prohibited. After the cecum was intubated, colonoscope withdrawal was performed exclusively with 2D or 3D images as per allocation. A standardized protocol for photo documentation of individual colonic segments and a withdrawal time of 6 minutes or longer were required during colonoscope withdrawal. During withdrawal, image-enhanced endoscopy (narrow-band imaging or chromoendoscopy with indigo carmine) was routinely used for suspicious lesions, and adenomas were removed/resected. The size, morphology, and location of each polyp were recorded, and specimens were sent for histological examination.

Therapeutic time was defined as the time for optical diagnosis and polyp removal. Mucosa inspection time was defined as withdrawal time minus therapeutic time. Incomplete colonoscopic examination was defined as failure to intubate the cecum or poor bowel preparation, and resulted in exclusion from the analysis. In line with the established clinical workflow of the participating institutions, bowel preparation was assessed with the modified Aronchick bowel preparation scale 21 22 .

### Outcomes

The primary outcome was ADR, defined as the proportion of patients with at least one adenoma detected during colonoscopy. Secondary outcomes were detection rates for flat (Paris classification 0-IIa, 0-IIb, or 0-IIc), sessile (Paris classification 0-Is), right-sided (cecum and ascending colon), left-sided (transverse colon to rectum), proximal (cecum to splenic flexure), distal (descending colon to rectum), and advanced adenomas, and sessile serrated lesions (SSLs). ADR stratified by size (<5 mm, 5–9 mm, ≥10 mm), polyp detection rate, mean adenoma number per patient, and mean polyp number per patient were also recorded. Advanced adenoma was defined as adenomas with size ≥10 mm, villous component, or high grade dysplasia according to World Health Organization classification 23 .

### Statistical analysis

A previous simulation study suggested that 3D colonoscopy could increase the ADR by 60% (from 42.7% to 67.7%) compared with standard colonoscopy 18 . Following international guidelines, we set the ADR with standard 2D colonoscopy at 25% 24 . To detect a 60% increase in ADR between 3D and standard 2D colonoscopy (40% vs. 25%) with an 80% statistical power and a two-sided significance level of 0.05, a minimum of 150 participants per group was needed. Accounting for potential exclusions or dropouts of approximately 10%, the enrollment target was at least 165 participants for each group. The analysis was by intention-to-treat.

Categorical variables were summarized using frequencies and percentages, and continuous variables as means and SDs. Statistical significance for categorical variables was tested using the Pearson chi-squared test, and differences between groups for continuous variables were tested using the independent sample *t* test. Univariable and multivariable logistic regression analyses were conducted to identify factors predictive of adenoma detection. Variables with a *P* value of <0.05 in the univariable analysis were included in the multivariable analysis, and variance inflation factor was used to detect multicollinearity. Post hoc analysis of the temporal changes in ADR and mucosa inspection time was conducted to explore the learning curve of 3D colonoscopy.

All analyses were performed using STATA software (StataCorp, College Station, Texas, USA). All tests were two-tailed, and differences were considered significant if *P* < 0.05.

## Results

### Patients

**Fig. 2:**
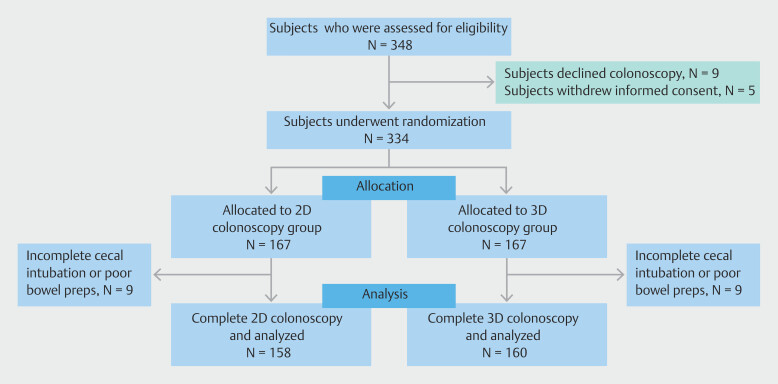
Screening, recruitment, randomization, and analysis of the study participants.

From February 2022 through June 2023, 348 individuals were screened for eligibility ( Fig. 2 ), and 339 consented to participate. Finally, 334 participants underwent colonoscopy and were randomly allocated to either the 2D or 3D group (n = 167 each). After excluding cases with incomplete colonoscopy and inadequate bowel preparation, 158 and 160 participants in the 2D and 3D groups, respectively, were analyzed. There was no crossover between the two groups.

### Baseline characteristics

The baseline characteristics and clinical information are summarized in Table 1 . Among the 318 enrolled participants, the mean age was 61.9 (SD 10.6) years and 150 (47.2%) were men. Most (69.8%) of the participants were asymptomatic, and the major indication for colonoscopy among the asymptomatic patients was positive fecal immunochemical test (FIT) or surveillance colonoscopy. The groups were comparable in age, sex, family history of CRC, smoking status, alcohol consumption, antithrombotic agent use, underlying diseases, colonoscopy indications, and bowel preparation status. There was no significant difference between the two groups in mucosa inspection time for all colonoscopies (2D vs. 3D: 9.4 [SD 3.1] vs. 9.8 [SD 2.6] minutes; *P* = 0.21).

**Table:** **Table 1** Demographics and clinical characteristics of study participants.

	2D colonoscopy (n = 158)	3D colonoscopy (n = 160)	*P*
Age, mean (SD), years	62.4 (11.2)	61.4 (9.9)	0.40
Male sex, n (%)	79 (50.0)	71 (44.4)	0.32
Body weight, mean (SD), kg	65.4 (12.2)	66.4 (13.2)	0.48
Body height, mean (SD), cm	164.2 (9.7)	162.9 (7.8)	0.19
Body mass index, mean (SD), kg/m ^2^	24.2 (4.2)	24.9 (4.1)	0.13
Family history of CRC, n (%)	17 (10.8)	27 (16.9)	0.11
Ever smoking, n (%)	39 (24.7)	32 (20.0)	0.32
Alcohol consumption, n (%)	14 (8.9)	11 (6.9)	0.51
Antithrombotic agent use, n (%)	21 (13.3)	27 (16.9)	0.37
Diabetes mellitus, n (%)	24 (15.2)	25 (15.6)	0.91
Hypertension, n (%)	52 (32.9)	64 (40.0)	0.19
Indication, n (%)			0.35
FIT positivity	44 (27.8)	50 (31.3)	
Post-polypectomy surveillance	59 (37.3)	45 (28.1)	
Symptoms	43 (27.2)	53 (33.1)	
Others	12 (7.6)	12 (7.5)	
Modified Aronchick bowel preparation scale, n (%) ^1^			0.09
Excellent/Good	100 (63.3)	87 (54.4)	
Fair	58 (36.7)	73 (45.6)	
Withdrawal time, mean (SD), minutes ^2^	11.0 (5.2)	12.5 (5.0)	0.009
Mucosa inspection time, mean (SD), minutes ^3^			
Entire cohort	9.4 (3.1)	9.8 (2.6)	0.21
Case numbers 1–40	9.6 (2.6)	11.1 (2.6)	0.01
Case numbers 41–80	10.0 (3.6)	10.1 (2.5)	0.90
Case numbers 81–120	9.5 (3.4)	9.9 (2.1)	0.54
Case numbers 121–160	8.3 (2.6)	8.0 (2.2)	0.64
2D/3D, two-dimensional/three-dimensional; CRC, colorectal cancer; FIT, fecal immunochemical test. ^1^ Participants rating poor or inadequate bowel preparation were excluded from the study. ^2^ Withdraw time = the total time from cecum to anus. ^3^ Inspection time = withdraw time – time for observing and removing polyps.			

### Outcomes

The 3D colonoscopy function was successfully implemented in all cases allocated to the 3D group without temporary equipment dysfunction during the colonoscopic procedures. For the two groups combined (n = 318), polyp detection rate and ADR were 54.4% and 45.9%, respectively. ADR was significantly higher in the 3D group compared with the 2D group (53.1% vs. 38.6%; difference 14.5% [95%CI 3.7 to 25.4]; odds ratio [OR] 1.80 [95%CI 1.15 to 2.82]; *P* = 0.009) ( Table 2 ). Regarding the secondary outcomes, compared with the 2D group, the 3D group had higher detection rates for flat adenomas (35.0% vs. 21.5%; difference 13.5% [95%CI 3.7 to 23.3]; OR 1.96 [95%CI 1.19 to 3.24]; *P* = 0.008), right-sided adenomas (26.3% vs. 15.2%; difference 11.1% [95%CI 2.2 to 19.9]; OR 1.98 [95%CI 1.14 to 3.48]; *P* = 0.02), proximal adenomas (38.1% vs. 23.4%; difference 14.7% [95%CI 4.7 to 24.7]; OR 2.02 [95%CI 1.24 to 3.28]; *P* = 0.005), and small (5–9 mm) adenomas (45.0% vs. 31.0%; difference 14.0% [95%CI 3.4 to 24.5]; OR 1.82 [95%CI 1.15 to 2.88]; *P* = 0.01). The median number of adenomas per patient was also higher in the 3D group (1 [interquartile range (IQR) 1–2] vs. 0 [IQR 0–1]; *P* = 0.03). As all individuals with adenomas had at least one left-sided adenoma (adenomas at transverse, descending, sigmoid colon, or rectum), the left-sided ADR was equivalent to the overall ADR in both groups. There was no significant difference in the detection rates for sessile adenomas, distal adenomas, advanced adenomas, and SSLs.

**Table:** **Table 2** Comparison of primary and secondary outcomes between two- and three-dimensional colonoscopy.

	Detection during colonoscopy		Difference in detection rate (95%CI), %	OR (95%CI)	*P*
	2D (n = 158)	3D (n = 160)			
Primary outcome, n (%)					
Adenoma	61 (38.6)	85 (53.1)	14.5 (3.7 to 25.4)	1.80 (1.15 to 2.82)	0.009
Secondary outcomes, n (%) ^1^					
Flat adenoma	34 (21.5)	56 (35.0)	13.5 (3.7 to 23.3)	1.96 (1.19 to 3.24)	0.008
Sessile adenoma	44 (27.8)	47 (29.4)	1.6 (–8.4 to 11.5)	1.08 (0.66 to 1.75)	0.76
Right-sided adenoma	24 (15.2)	42 (26.3)	11.1 (2.2 to 19.9)	1.98 (1.14 to 3.48)	0.02
Left-sided adenoma	61 (38.6)	85 (53.1)	14.5 (3.7 to 25.4)	1.80 (1.15 to 2.82)	0.009
Proximal adenoma	37 (23.4)	61 (38.1)	14.7 (4.7 to 24.7)	2.02 (1.24 to 3.28)	0.005
Distal adenoma	48 (30.4)	53 (33.1)	2.7 (–7.5 to 12.8)	1.11 (0.68 to1.82)	0.66
Sessile serrated lesions	8 (5.1)	11 (6.9)	1.8 (–3.4 to 7.0)	1.38 (0.54 to 3.54)	0.50
Advanced adenoma	11 (7.0)	15 (9.4)	2.4 (–3.6 to 8.4)	1.38 (0.61 to 4.11)	0.43
Adenoma, n (%)					
<5 mm	14 (8.9)	13 (8.1)	0.7 (–6.9 to 5.4)	1.10 (0.50 to 2.42)	0.81
5–9 mm	49 (31.0)	72 (45.0)	14.0 (3.4 to 24.5)	1.82 (1.15 to 2.88)	0.01
≥10 mm	15 (9.5)	25 (15.6)	6.1 (–1.1 to 13.4)	1.77 (0.89 to 3.49)	0.10
Polyps, n (%)	73 (46.2)	100 (62.5)	16.3 (5.5 to 27.1)	1.94 (1.24 to 3.04)	0.004
Adenomas per patient, median (IQR), n	0 (0–1)	1 (1–2)	–	–	0.03
Polyps per patient, median (IQR), n	0 (0–1)	1 (0–2)	–	–	<0.001
2D/3D, two-dimensional/three-dimensional; IQR, interquartile range; OR, odds ratio. ^1^ Right-sided adenoma: adenoma at cecum or ascending colon; left-sided adenoma: adenoma at transverse colon, descending colon, sigmoid colon, or rectum; proximal adenoma: adenoma at cecum, ascending colon, or transverse colon; distal adenoma: adenoma at descending colon, sigmoid colon, or rectum; flat adenoma: Paris classification 0-IIa, 0-IIb, or 0-IIc; sessile adenoma: Paris classification 0-Is.					

### Factors associated with adenoma detection

In the univariable logistic regression analysis, age, hypertension, FIT positivity, bowel preparation (excellent/good vs. fair), mucosa inspection time, and 3D colonoscopy were significantly associated with adenoma detection ( Table 3 ). The multivariable analysis showed that 3D colonoscopy was independently associated with adenoma detection (adjusted OR [aOR] 1.76 [95%CI 1.09 to 2.83]) after adjusting for FIT positivity, mucosa inspection time, and other confounders. Age (aOR 1.03 [95%CI 1.01 to 1.06]) and mucosa inspection time (aOR 1.16 [95%CI 1.06 to 1.28]) were also independently associated with adenoma detection.

**Table:** **Table 3** Factors associated with detection of adenoma.

	Univariable analysis		Multivariable analysis	
	OR (95%CI)	*P*	aOR (95%CI)	*P*
Age, per 1-year increment	1.04 (1.02 to 1.06)	<0.001	1.03 (1.01 to 1.06)	0.008
Male sex	1.44 (0.92 to 2.24)	0.11		
BMI, per 1-kg/m ^2^ increment	1.04 (0.98 to 1.10)	0.16		
Ever smoking	1.70 (1.00 to 2.90)	0.05		
Alcohol consumption	1.30 (0.57 to 2.93)	0.54		
Antithrombotic agent use	1.79 (0.96 to 3.34)	0.07		
Diabetes mellitus	1.40 (0.76 to 2.58)	0.28		
Hypertension	1.90 (1.20 to 3.02)	0.006	1.32 (0.79 to 2.20)	0.29
Family history of CRC	0.63 (0.33 to 1.21)	0.17		
FIT positivity	1.90 (1.17 to 3.10)	0.01	1.46 (0.86 to 2.47)	0.16
Good/excellent bowel preparation	0.60 (0.38 to 0.94)	0.03	0.89 (0.54 to 1.47)	0.67
Mucosa inspection time, per 1-minute increment	1.20 (1.10 to 1.31)	<0.001	1.16 (1.06 to 1.28)	0.001
3D colonoscopy use	1.80 (1.15 to 2.81)	0.009	1.76 (1.09 to 2.83)	0.02
3D, three-dimensional; (a)OR, (adjusted) odds ratio; BMI, body mass index; CRC, colorectal cancer; FIT, fecal immunochemical test.				

### Temporal changes in ADR and mucosa inspection time

**Fig. 3:**
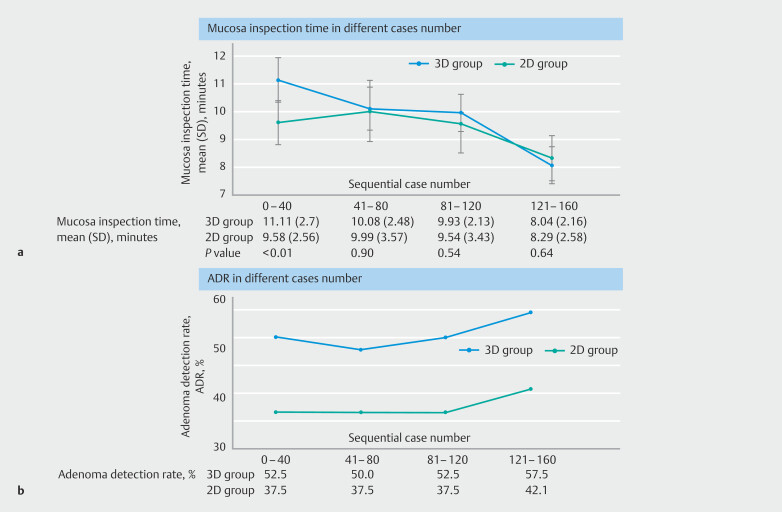
Temporal changes between two- and three-dimensional colonoscopy. **a** Mean mucosa inspection time. **b** Adenoma detection rate. 2D/3D, two-dimensional/three-dimensional.

Compared with the 2D group, the mean mucosa inspection time in the 3D group was significantly longer in the first 40 examinations (11.1 [SD 2.6] vs. 9.6 [SD 2.6] minutes; *P* = 0.01) but became comparable thereafter ( Table 1 , Fig. 3 **a** ). Similar trends were observed for each endoscopist, with interendoscopist variations. The learning curve, as inferred by the difference in mucosa inspection time between 3D and 2D colonoscopy, seemed shortest for the senior colonoscopist, with the time difference reduced from 2.8 minutes for procedures 1–10 to 0.5 minutes for procedures 11–20. By contrast, one junior endoscopist appeared to have the longest learning curve (time difference 1.9, 0.9, and 0.5 minutes for procedures 1–10, 11–20, and 21–30, respectively). On the other hand, ADR in the 3D group was consistently higher than that in the 2D group by approximately 15% throughout the study, even among the first 40 examinations ( Fig. 3 **b** ). All endoscopists achieved numerically higher ADRs with 3D colonoscopy (difference in ADRs [3D minus 2D]: senior endoscopist 12%; junior endoscopists 12.5%, 21.6%, and 50.0%, respectively). However, per-endoscopist analyses on differences in mucosa inspection time and ADR were post hoc and included a limited sample size, and thus should be considered exploratory.

## Discussion

This RCT conducted in individuals aged 40 years or older showed that 3D colonoscopy resulted in a significant 15% increase in ADR, as well as increases in the detection rates of small, flat, right-sided, and proximal neoplasms, which are commonly overlooked by standard 2D colonoscopy. Notably, 3D colonoscopy enhanced polyp detection without increasing the mucosa inspection time and could be used in conjunction with other image-enhancing modalities such as narrow-band imaging and chromoendoscopy.

Enhancing the ADR is crucial for reducing the incidence of PCCRC and associated mortality 5 . Despite the multitude of advanced image-processing technologies that have been developed to improve adenoma detection 6 , the incidence of PCCRC remains as high as 8% in Asia and Europe and is mainly attributed to missed neoplasms during colonoscopy 11 25 26 . Neoplasms with flat morphology, particularly those located in the proximal colon, are more likely to be overlooked 27 . The larger colonic folds in the proximal colon, where neoplasms are more often flat, further compound adenoma detection 28 . This study corroborated the notion that 3D colonoscopy enhances anatomical details and depth perception and thereby facilitates identification of those hard-to-detect neoplasms. Our finding that 3D colonoscopy improved ADR and detection of flat, right-sided, and proximal adenomas, respectively, support its potential to reduce PCCRCs, warranting further long-term follow-up research. Multicenter clinical trials and real-world studies, advocacy by gastroenterology societies and opinion leaders, regulatory approval, and education/training are crucial for the dissemination of 3D colonoscopy.

The finding that 3D colonoscopy mainly enhanced the detection of small polyps (5–9 mm) might be due to these polyps being near the threshold of detection with 2D colonoscopy; therefore, enhanced depth perception conferred by 3D colonoscopy significantly increased the ability to detect these polyps. By contrast, polyps 1–5 mm might remain difficult to detect despite enhanced depth perception and thus 3D colonoscopy did not significantly improve detection. In line with this notion, studies on chromoendoscopy using indigo carmine found no or minimal improvement in detecting adenomas 1–5 mm 29 30 . On the other hand, polyps >10 mm could be easily detected by 2D colonoscopy, with limited room for further improvement by 3D colonoscopy.

It is worth noting that while high ADRs (ADR 38.6%, right-sided ADR 15.2%, proximal ADR 23.4%, flat ADR 21.5%) were achieved by standard 2D colonoscopy with a mean mucosa inspection time of approximately 9 minutes, 3D colonoscopy could further increase the ADRs by approximately 15% (ADR 53.1%, right-sided ADR 26.3%, proximal ADR 38.1%, flat ADR 35.0%). The ADRs of the 2D group in our study were in line with a recent RCT by Zhao et al., which showed that 2D white-light colonoscopy with a mucosa inspection time of 9 minutes achieved ADR, proximal ADR, and flat ADR of 36.6%, 21.4%, and 27.4%, respectively 31 . An OR of 1.76 for detecting adenomas after adjusting for mucosa inspection time and other confounders firmly supported that 3D colonoscopy provided a distinct advantage over 2D colonoscopy in adenoma detection that cannot be provided by alternative means such as increasing the mucosa inspection time. Whether 3D colonoscopy could provide greater benefit over standard 2D colonoscopy in real clinical settings where the mucosa inspection time is shorter than 9 minutes warrants further study.

Our exploratory analysis confirmed that 3D colonoscopy has a short learning curve and consistently confers an improvement in ADR even during the learning phase. The findings suggested a learning curve of between 10 and 20 procedures for 3D colonoscopy, with interendoscopist variation. Taken together, the consistent benefit in ADR and short learning curve suggest that 3D colonoscopy could be easily adopted by endoscopists in routine colonoscopy practice.

A recent crossover RCT including patients younger than 40 years compared 2D then 3D vs. 3D then 2D colonoscopy (i.e. tandem colonoscopy) and showed that ADR in the first examination was comparable between 3D and 2D colonoscopy (24.7% vs. 23.8%), whereas in the second examination ADR was significantly higher with 3D compared with 2D (13.8% vs. 9.9%) 32 . However, the tandem colonoscopy design could introduce bias, because the diagnostic performance of the second examination is influenced by the findings of the first one. In contrast, the parallel design of the current study minimized bias, better reflected clinical reality, and used ADR, the surrogate for PCCRC, as the primary outcome. Notably, the ADR of the first colonoscopy in the previous study did not differ between 2D and 3D and seemed lower than that in the current study, probably due to the shorter withdrawal time (<6 minutes) and the inclusion of younger patients (aged 18–40 years) in that study. In contrast, the current study enrolled individuals aged over 40 years, and thus the results should be more generalizable to the examinees of clinical colonoscopy practice, and the ability to further improve ADR where colonoscopy quality assurance measures were rigorously implemented highlighted the benefit of 3D colonoscopy in enhancing adenoma detection. The use of different 3D endoscopy systems could have also contributed to the differences between the two studies, as the vividness of 3D visualization might differ between systems, depending on the image reprocessing algorithms employed.

This study had several notable strengths. This RCT is the first to demonstrate the ability of 3D imaging to improve ADR and enhance detection of flat and proximally located adenomas, which are challenging to detect with standard 2D colonoscopy. Second, this study ensured high-quality colonoscopy through measures such as attention to bowel cleansing, photo documentation, and maintaining a withdrawal time exceeding 6 minutes, in accordance with the international benchmarks. Third, this study enrolled individuals aged over 40 years to align the study population with the examinees in general colonoscopy practice, enhancing the relevance and generalizability of the results. Finally, this study conducted stratified comparisons according to polyp morphologies and location, revealing the advantage of 3D colonoscopy in enhancing detection of flat and proximal adenomas.

This study also had limitations. Given the apparent differences between 2D and 3D colonoscopy, it was not possible for the colonoscopists to be blinded to group allocation. However, the quality assurance program, including standardized photo documentation in participating institutions, ensured that the mucosa inspection time was comparable between the two groups and >6 minutes, refuting the possibility that colonoscopists tried harder to find polyps in the 3D group. Therefore, nonblinding of endoscopists should not have introduced significant bias. While the endoscopists’ ADRs might have been affected by study participation (i.e. Hawthorne effect), the potential influence should occur in both 2D and 3D groups to a similar degree; therefore, the observed differences in ADR should be little influenced by the Hawthorne effect and remain valid. The comparability in other procedural factors and randomization minimized the possibility of confounding, and regression analysis adjustment for potential confounders further confirmed that the observed improvement in adenoma detection was attributed to 3D colonoscopy. Second, given the limited availability of the newly developed 3D colonoscopy equipment, this RCT included only a limited number of institutions and colonoscopists. A larger trial including more institutions/colonoscopists and diverse patient populations is warranted to further ascertain the potential benefit conferred by wide implementation of 3D colonoscopy. Third, this study did not evaluate the endoscopists’ burden such as eye strain because of the lack of a well-established objective evaluation tool/method. However, none of the participating endoscopists reported fatigue or eye strain after performing 3D colonoscopy, probably because this 3D endoscopy system uniquely considers pupillary distance. Tailoring the 2D to 3D conversion process according to pupillary distance is crucial for mitigating visual discomfort when watching 3D imagery 33 . The finding that a significant increase in ADR with 3D colonoscopy was not accompanied by an increase in the mucosa inspection time compared with 2D colonoscopy also suggest that processing the 3D images did not significantly increase endoscopist burden. Whether more prolonged use of this 3D system for colonoscopy might increase endoscopist burden remains to be evaluated. Finally, given the relatively low prevalence of SSLs and advanced adenomas, this study was not powered to detect potential differences in the rate of SSLs and advanced adenomas between 3D and 2D colonoscopy. The numerically higher detection rates for SSLs and advanced adenomas with 3D colonoscopy observed in this study warrants confirmation by further research with larger sample sizes.

In conclusion, this RCT demonstrated that for individuals aged 40 years and above, 3D colonoscopy significantly increased the detection rates for adenomas, particularly small, flat, and proximal adenomas, compared with standard 2D colonoscopy. The sizable increases in ADR suggest that implementing 3D colonoscopy in clinical practice might deliver significant improvement in patient outcomes.
